# Optimizing and characterization of titanium dioxide extracted from black sand using response surface methodology (RSM)

**DOI:** 10.1186/s13065-025-01675-z

**Published:** 2025-12-01

**Authors:** Sohair T. Aly, Sabah Mohamed Farouk, Hossam Zaki, Tohamy A. Tohamy, Mahmoud E. Sheikh, Abd El Rhman Z. Sultan, Mustafa Fouda, Hisham Mustafa, Omar Abdel Sater, Merna Ezzat, Mahmoud A. Mabrouk

**Affiliations:** Chemical Engineering Department, Egyptian Academy for Engineering and Advanced Technology (EA&EAT), Affiliated to the Ministry of Military Production, Km. 3 Cairo Belbeis Desert Rd., Cairo Governorate, 3066 Egypt

**Keywords:** Black sand, Illeminite, Titanium dioxide, Leaching, Hydrolysis

## Abstract

Titanium dioxide is used in different applications such as paints, plastics, paper, and water treatment. Various routes are applied for the recovery of TiO_2_ according to the available raw material. The aim of this work is the application of response surface methodology for optimizing the recovery of TiO_2_ from illeminite beach sand deposits (black sand, Kafr-El-Sheikh, Egypt) with high purity using sulfuric acid. Utilizing local raw material to obtain titanium dioxide with high purity for applications in paints and energy applications. Applying a simple method for separating iron, which is present in high weight% (49%). Application of the RSM approach in the extraction of titanium dioxide to determine the interaction of the studied parameters and determine the model relating them to the yield of titanium dioxide. characterizations of raw material are applied; X-ray fluorescence (XRF) indicates the chemical composition, and the weight% of titanium dioxide is 43%. The illeminite phase is confirmed by X-ray diffraction (XRD) of the raw material sample. The average particle size of illeminite powder is measured using screen analysis, and it is around 118 micrometers. Three selected parameters affecting the leaching of illeminite are studied using the Response Surface Methodology (RSM) technique: temperature (60–120℃), sulphuric acid concentration (4–12 M), and time (1–5 h). Maximum conversion (84%) is obtained at the optimum conditions predicted by the RSM model at Temperature (120 ℃), time 4.2 h, Concentration (12 M). The relation between the studied parameters and conversion is represented by a reduced cubic model with R^2^ = 0.973 and p-value = 0.0001, which confirms the accuracy of the model. The prepared titanium dioxide is separated from the filtrate solution by hydrolysis at 109 °C for 3 h, using EDTA as a complexation agent to capture the solvated iron. The hydrated titanium dioxide powder is dried, then calcinated at 500 °C for 3 h to produce titanium dioxide in an anatase phase. The prepared sample is examined using XRF, XRD, and SEM-EDX. 96.4% purity of titanium dioxide is formed; the XRD pattern of the examined sample confirmed the formation of the anatase phase. SEM images display that TiO_2_ has a uniform spherical morphology. The EDX spectrum shows that most of the sample is TiO_2_. The average particle size of the prepared titanium dioxide using a particle size analyzer is about 78.82 nm.

## Introduction

### Background

Titanium containing compounds are used in many applications, such as environmental remediation, energy technologies, the pharmaceutical industry, the paint industry, Photocatalysis, and the textile industry. The ability to produce titanium species from their ore materials is highly significant in the field of research as well as the industrial sector [1–10].

In recent years, titanium has become an essential commodity. The primary economically significant titanium oxides include ilmenite (FeTiO_3_) and the polymorphs of titanium dioxide (TiO_2_) – rutile, anatase, and brookite. Perovskite (CaTiO_3_) and titanite (CaTiSiO_5_) are less common sub-economic minerals [3].

Ilmenite, separated from black sands, can be used as an alternative source of raw material for the production of TiO_2_ [11, 12]. Ilmenite contains 30% to 65% TiO_2_. More than half of the world’s titanium production is obtained from ilmenite and rutile [13].

Among the iron-titanium-oxide ore minerals, titaniferous magnetite contains 1 to 15% titanium, while titanomagnetite contains 2 to 20% titanium [3]. TiO_2_, or inorganic titanium dioxide, is a stable and non-toxic material. Given its extraordinarily high refractive index, TiO_2_ has been widely used as a white pigment. For pigment applications, TiO_2_ particles should have a size of approximately 250 nm. Titanium dioxide is used in many commonplace products, such as paints, plastics, paper, cosmetics, and more [3].

### Applications

In 2022, the paints and coatings category held a dominant position in the market, contributing over 59.3% of worldwide sales. The fastest increase in the segment is expected to occur between 2023 and 2030. When added to coatings, its extremely effective properties of dispersing visible light produce whiteness, brightness, and opacity. Over the past few years, the paints and coatings industry’s product demand has been driven by significant expansion in the building and automotive industries, particularly in developing nations [14].

One abundant, clean, and sustainable energy source is solar energy. Nowadays, the most widely used method of converting sunlight into electrical power is through solid-state solar cells, which are based on silicon. Recently, new research on solar cells based on semiconducting materials has produced an alternative photovoltaic technology with the promise of flexible and inexpensive manufacturing. Nano-structured TiO_2_ is the main semiconducting element in these latest solar cell generations. This approach uses an electron sensitizer that absorbs the visible to inject charge carriers across the semiconductor-electrolyte junction into TiO_2_ since TiO_2_ only absorbs the ultraviolet portion of solar radiation with its band gap of 3.2 electron volts (eV) [14–17].

Lithium-ion rechargeable batteries are commonly seen in consumer electronics. Lithium-ion battery systems and technologies have revolutionized the power supply battery market. Anode materials based on titanium oxides are the most promising options to replace carbonaceous anodes because of their advantages in terms of cost, safety, and toxicity. TiO_2_ also has a high plateau discharge voltage, outstanding cycle stability, and superior structural stability when compared to Li+/Li [14].

Another important application of nano-structured TiO_2_ is in the water treatment business, which takes advantage of its photocatalytic properties. The application of nano-structured TiO_2_ for water filtration has been the subject of intense research in recent years. TiO_2_ has been used in the photocatalytic degradation of organic contaminants in wastewater, as well as pesticides, dyes, and medications in other contaminated water [4, 14].

TiO_2_ is a semiconducting material with an extremely high refractive index. The material can scatter visible light because of its high refractive index. The present method of guarding against harmful UV radiation involves suspending a substance, like sunscreen, that either scatters or absorbs UV radiation in a thick emulsion. Sunscreens typically contain titanium dioxide (TiO_2_) at a concentration of 2–15%. Physical filters, such as ZnO and TiO_2_, which block UVA and UVB light via absorption, reflection, and scattering, and chemical filters, which are organic compounds that strongly absorb UV, are both frequently found in sunscreen [14, 18].

The market for titanium dioxide was estimated to be worth USD 18.82 billion in 2022, and between 2023 and 2030, it is projected to expand at a compound annual growth rate (CAGR) of 6.3%. Due to the overuse of paints and coatings in a variety of end-use industries, such as the construction and automotive industries, the market is anticipated to develop significantly. The growing need for titanium dioxide (TiO_2_) from end-user industries is responsible for the market’s expansion. The global market for pigments is being driven primarily by rising demand from a variety of end-use sectors, including paints and coatings, textiles, printing inks, plastics, and others [19].

### The extraction methods

Titanium dioxide can be extracted from ilmenite through a different process, such as acidic leaching by using hydrochloric acid or sulfuric acid, or alkaline decomposition is also used to extract ilmenite (i.e., by using sodium hydroxide). There are also some desirable and undesirable elements present in ilmenite, which depend on the type and location of the raw material [20–22].

The significant effects of reaction temperature, KOH concentration, stirring speed, particle size, and alkali-to-ilmenite mass ratios on titanium extraction were studied by LIU, Yumin, et al. in 2006. Approximately 80–85% of the titanium could be leached from the ilmenite ore under the optimal conditions [20].

GINTING, Lavita Indriani Br, et al. [23] used caustic fusion, acid leaching using hydrochloric acid, and the use of citric acid were conducted. Caustic fusion is conducted with a ratio between ilmenite and NaOH is 1:2, with a fusion temperature of 850 °C and a fusion time of 60 min. The next process is water leaching and acid leaching. The result of this study is that the TiO_2_ obtained from each acid leaching is hydrochloric acid leaching, producing powder pigment with 94.189% TiO2, acetic acid leaching producing 37.099% TiO_2_, citric acid leaching producing 41.480% and hydrochloric acid leaching without fusion process producing 43.991% TiO_2_.

XIONG, Xunhui, et al. [24] obtained the high-purity TiO_2_, by introducing ethylenediaminetetraacetic acid (EDTA) as an additive to suppress the precipitation of Fe^3+^ during the hydrolysis of the titanium sulfate solution.

The mineral ilmenite is mined from black sand, which is found in vast amounts in several locations around Egypt [25–27]. The Nuclear Materials Authority conducted an aerial survey that revealed 11 locations along Egypt’s northern coast with black sand. The geological reserve of those sands on the Egyptian coast, including the Rasheed area with 600 million cubic meters of reserves, the Damietta area with 300 million cubic meters, and the Arish Rafah area with 200 million cubic meters, is estimated to be around 1.3 billion cubic meters based on studies and research [28].

### The objectives of the study

The present work is directed to the application of a statistical model (RSM approach, Box-Behnken statistical model is applied) for optimizing the parameters affecting the extraction of titanium dioxide from ilmenite extracted from black sand using sulphuric acid to maintain maximum possible yield. Efficient separation of iron using a suitable method, as it is present in high percentages in the raw material.

Experimental Statistical Design-Expert 12, software was used to apply the experimental Box-Behnken statistical design in 3^3^ factorial design as a tool for simulation and optimization of the dissolution process. As second-order model was selected that relates the target variable Y to the three independent variables X_1_, X_2_ and X_3_ by the relation:

1 $$\begin{array}{l}{\rm{Y}} = {\beta _0} + {\beta _1}{X_1} + {\beta _2}{X_2} + {\beta _3}{X_3} + {\beta _{12}}{X_1}{X_2} + {\beta _{23}}{X_2}{X_3}\\ \quad + {\beta _{31}}{X_3}{X_1} + {\beta _{11}}{X_{12}} + {\beta _{22}}{X_{22}} + {\beta _{33}}{X_{32}} \end{array}$$

Besides disclosing the values of the constants _0_, _1_,_2_ …, the software enables drawing two- and three-dimensional plots for response surfaces. In this selected method, fewer runs are required, especially for three or four factors. This translates directly to reduced time, material consumption, and overall experimental cost [29].

There are different design techniques that can be applied, such as; Taguchi experimental design technique. It is an efficient technique that ensures excellent performance in the design of processes or products. Despite the advantage of Taguchi experimental design, it can only optimize a singular performance characteristic, which limits it for optimize multiple performance characteristics [30–32].

RSM is selected as it is a powerful, statistically rigorous, and cost-effective method for modeling, analyzing, and optimizing industrial and scientific processes when the relationship between factors and the response is expected to be smooth and moderately complex. For machine learning or AI-based predictive optimization process ML/AI, a high-quality, non-linear model may require hundreds or thousands of data points to generalize well and avoid overfitting. If only sparse experimental data is available, ML/AI often performs poorly [33].

## Materials and methods

### Materials

Ilmenite beach sand deposits were obtained from the Egyptian company for black sand. Concentrated Sulfuric acid (98%), and disodium salt of ethylenediaminetetraacetic acid (EDTA). were purchased from the Middle East Chemicals company.

### Method

#### Leaching

A weighted sample of ilmenite (50 g) is leached with diluted sulfuric acid (250 g) and placed in a 500 cm^3^ glass conical flask glass beaker associated magnetic stirrer, and a condenser.

**Fig. 1:**
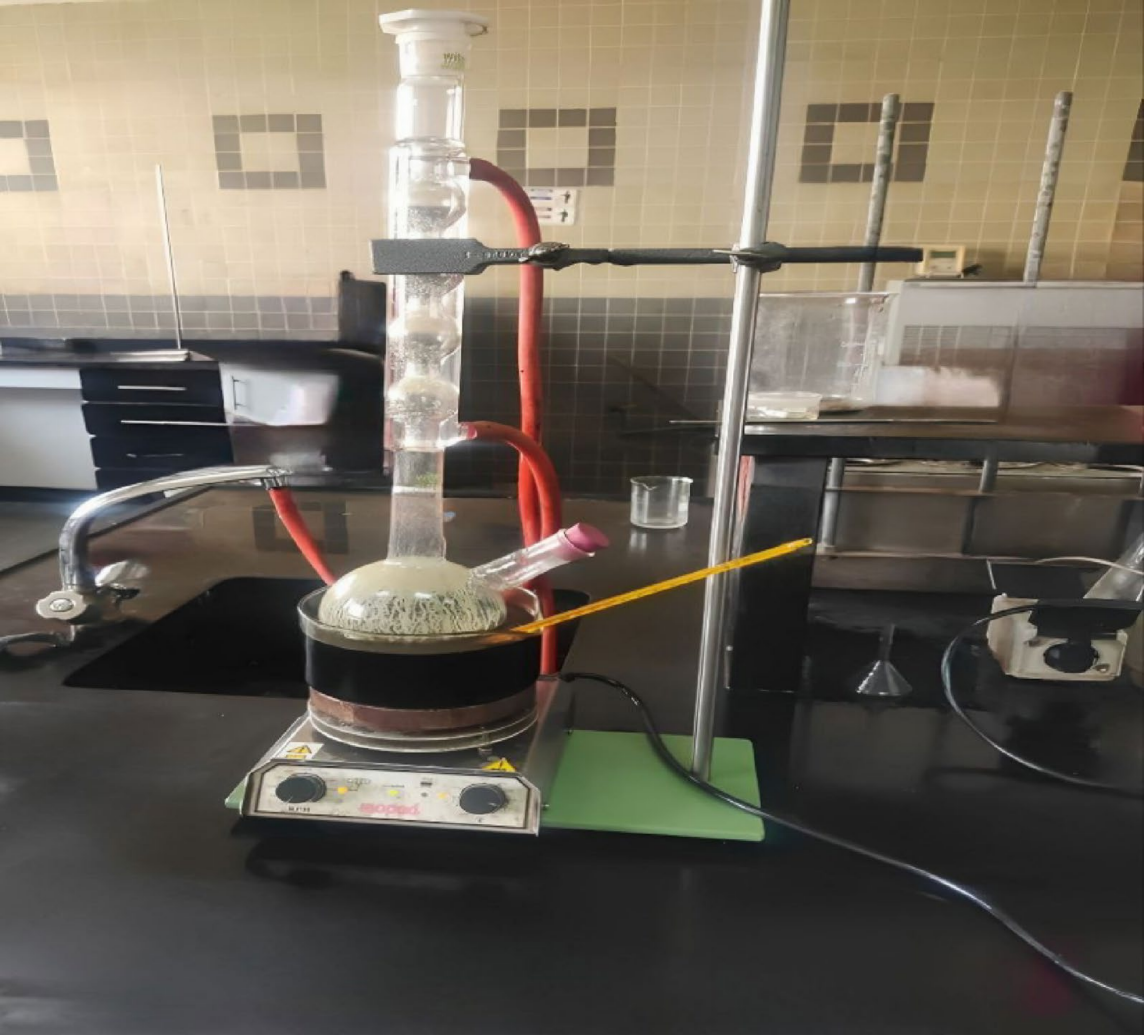
Setup of the leaching process

Sulfuric acid was poured into the reactor along with the ilmenite sample and heated to the desired temperature using a water path or oil path according to the reaction temperature. The temperature was measured by a thermometer during the reaction. Stirring speed is fixed at (250 rpm) and solid to liquid ratio is at 1:5 for all runs [3, 34]. Figure 1, shows the reaction setup of the leaching process.

The reaction can be represented by the following equation:

1 $$\begin{array}{l}{\rm{2}}{{\rm{H}}_{\rm{2}}}{\rm{S}}{{\rm{O}}_{\rm{4}}}\left( {{\rm{aq}}} \right) + {\rm{FeTi}}{{\rm{O}}_{\rm{3}}}\left( {\rm{s}} \right) \to {\rm{TiOS}}{{\rm{O}}_{\rm{4}}}\left( {{\rm{aq}}} \right)\\\quad + {\rm{2}}{{\rm{H}}_{\rm{2}}}{\rm{O }}\left( {\rm{l}} \right) + {\rm{FeS}}{{\rm{O}}_{\rm{4}}}\left( {{\rm{aq}}} \right)\end{array}$$

The solution is filtered to remove the unreacted ilmenite.

### Experimental design and data analysis

The studied parameters temperature, reaction time, and acid concentration, are optimized by using response surface methodology (RSM, Box-Behnken Design) [29]. Experimental conditions are displayed in Table 1. To maximize the response (conversion) and assess the impact of the independent process variables, a response surface approach based on Box-Behnken experimental design was used with five runs at the center point and a total of 17 experimental runs (time: 1–5 h; temperature: 60–120 0 C; Acid concentration: (4–12 M). The statistical analysis, modeling, and optimization were done with Experimental Statistical Design-Expert 12 software.

**Table 1:** Experimental conditions for the leaching process

Variable	Min	Center	Max
Temperature of reaction (°C)	60	90	120
Acid concentration (M)	4	8	12
Time of reaction (h)	1	3	5

#### Hydrolysis

**Fig. 2:**
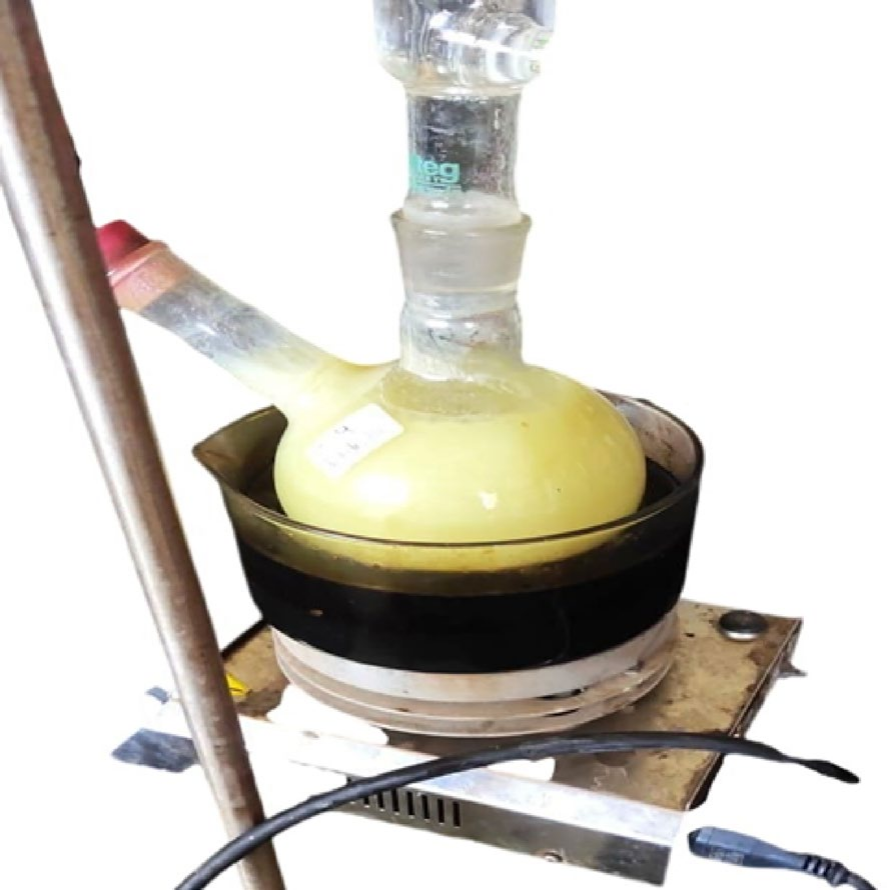
Hydrolyses of filtrate solution

The filtrate solution is hydrolyzed using water with a volume equal to 3 times its volume. The titanium sulfate is hydrolyzed by placing it into a 500 cm^3^ glass conical flask glass beaker associated with a magnetic stirrer and condenser Fig. 2. 1 ml of 1 molar EDTA is added to 20 ml of filtrate solution. EDTA is added as a complexing agent to prevent precipitation of iron hydroxide (Fe(OH)₃). The hydrated titanium dioxide solution is headed to 109 °C for 3 h. White precipitate of hydrated titanium dioxide is formed at the bottom. The precipitate is washed several times with distilled water to remove any residual of sulphuric acid. The precipitate is separated, then dried at 100 °C for about 8 h [3, 34].

2 $${\text{TiOS}}{{\text{O}}_{\text{4}}}\left( {{\text{aq}}} \right)+{\text{2}}{{\text{H}}_{\text{2}}}{\text{O}} \to {\text{TiO}}{\left( {{\text{OH}}} \right)_{\text{2}}}\left( {\text{s}} \right)+{{\text{H}}_{\text{2}}}{\text{S}}{{\text{O}}_{{\text{4}}({\text{aq}}}})$$

#### Calcination

The dried product (TiO_2_ nH_2_O, hydrous titania) is calcined in an electric furnace, at temperatures of 500 °C for 4 h.

3 $${\text{TiO}}{\left( {{\text{OH}}} \right)_{\text{2}}}\left( {\text{s}} \right) \to {\text{Ti}}{{\text{O}}_{\text{2}}}\left( {\text{s}} \right)+{{\text{H}}_{\text{2}}}{\text{O}}$$

## Results and discussion

The following sections present and discuss the characterization of raw material, product, and the results of the applied statistical model. Screen analysis is applied for ilmenite powder to determine the average grain size, X-ray fluorescence (XRF), X-ray diffraction (XRD), and inductively coupled plasma (ICP); these are used for the characterization of raw materials and prepared samples [35, 36].

### X-ray fluorescence

X-ray Fluorescence (XRF) analysis is used to determine the elemental composition of materials. The Panalytical 2005, WD-XRF spectrometer of type AXIOS was used.

The XRF results of the ilmenite sample, as shown in Table 2 : XRF analysis result state that the sand is mainly composed of iron (49.054% Fe_2_O_3_), titanium (43.127% TiO_2_), and traces of different materials such as magnesium and aluminum oxides [1].

**Table 2:** XRF analysis result

Compound formula	Percentage (wt%)
Na_2_O	0.173
MgO	1.004
Al_2_O_3_	1.034
SiO_2_	3.165
P_2_O_5_	0.105
SO_3_	0.038
K_2_O	0.050
CaO	0.506
TiO_2_	43.127
Cr_2_O_3_	0.197
MnO	1.313
Fe_2_O_3_	49.054
Co_3_O_4_	0.079
ZnO	0.028
ZrO_2_	0.069
Nb_2_O_5_	0.033
Cl	0.025

### X-ray diffraction

**Fig. 3:**
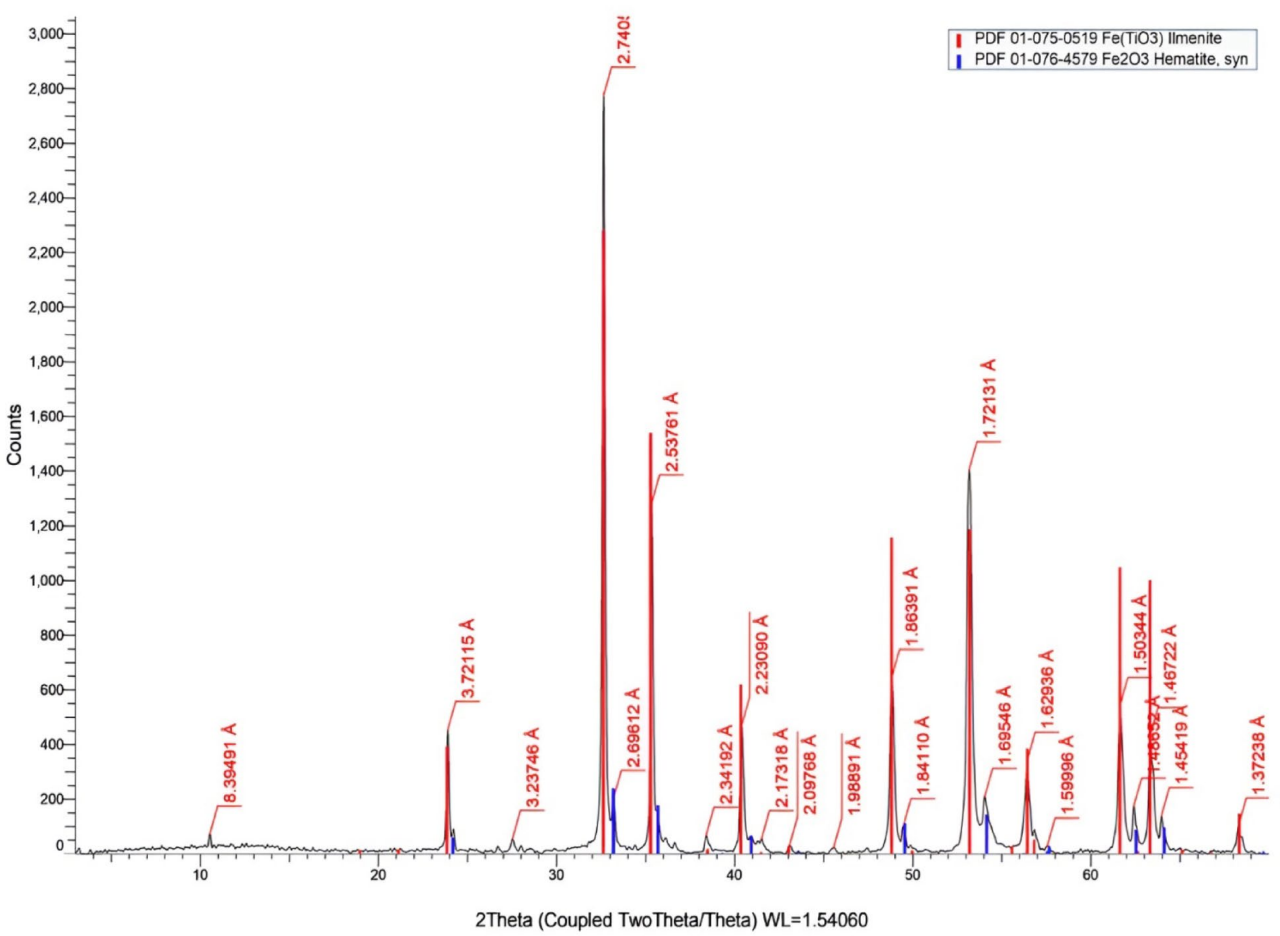
XRD of illeminite sample

X-ray diffraction is used to determine the crystal structure of a raw material sample. Mono-achromatized Copper Kα radiation was used for X-ray diffraction (XRD) at 40 mA and 40 kV. A kind of Philips diffractometer (PW 1730) was utilized, scanning at a rate of 2º/min B (Ni-filtered Cu Kα radiation). X-ray diffraction (XRD) results indicated that the main phase of the sample is ilmenite (89.7%) as shown in Fig. 3; Tables 1 and 30.3% is hematite, which is compatible with the results obtained in XRF analysis of the sample [37].

**Table 3:** XRD results

Color	Compound name	Formula	Percentage
	Ilmenite	Fe(TiO_3_)	89.7%
	Hematite, syn	Fe_2_O_3_	10.3%

### Optimization of leaching parameters using RSM

Leaching runs were carried out according to the experimental design obtained from the Box-Behnken design. After the leaching process, inductively coupled plasma was used to detect the concentration of Ti ions in the filtrate solution obtained from the leaching step. It measures the concentration of elements using the de-excitation of atoms and ions in a plasma. ICP OES – PLASMA 3000 was used [38].

The conversion of the leaching reaction was calculated according to the ICP analysis, and Table 4 displays the result of the conversion for different runs at different conditions, as seen in the next table. Experiment number 7 has the highest conversion value (77.68%) at 120 °C, 12 M, and 3 h. Experiment number 1 has the lowest conversion (0.287%) at 60 °C, 8 M, and 1 h.

Conversion was calculated according to Eq. 4 [39]

4 $$\begin{array}{l}Conversion = \\ \frac{{\frac{{Concentration~of~titanium~\left( {PPM} \right)}}{{1000}} \times Liquid~volume~of~sample\left( {ml} \right)}}{{12.9~ \times 1000}}\\ \times 100\end{array}$$

**Table 4:** Actual experimental results with different parameters

Run	Factor 1 A: Temperature (℃)	Factor 2 B: Concentration (M)	Factor 3 C: Time (h)	Response conversion (%)
1	60	8	1	0.29
2	60	12	3	7.93
3	90	12	5	32.68
4	120	8	5	43.65
5	120	4	3	73.34
6	90	4	5	8.66
7	120	12	3	77.68
8	90	4	1	1.74
9	60	4	3	1.3
10	90	8	3	9.38
11	90	8	3	11.64
12	120	8	1	17.06
13	90	8	3	10.24
14	90	8	3	3.87
15	90	12	1	8.15
16	60	8	5	1.80
17	90	8	3	19.28

### Analysis of variance (ANOVA)

According to the data obtained from actual experimental results with different parameters, as shown in Table 4, the relation between the response ( y; conversion of titanium) and the three selected parameters is fitted to a reduced cubic model. The validity of the model was verified through different tests, the confidence level (p-value), Fisher test (F-value) and R^2^, and adjusted R^2^. The Fisher test and p-value test were also applied on each term in the model to determine if it affects the conversion or can be neglected.

The results were fitted to a reduced cubic model, with R^2^ of 0.973, adjusted R^2^ of 0.9385, and P value is 0.0001, which indicates the model is significant. All the statistical results are shown in Table 5.

**Table 5:** ANOVA for reduced cubic model

Source	Sum of squares	df	Mean square	F-value	*p*-value	
Model	9000.09	9	1000.01	28.11	0.0001	Significant
A-Temperature	859.27	1	859.27	24.15	0.0017	
B-Concentration	214.35	1	214.35	6.02	0.0438	
C-time	443.41	1	443.41	12.46	0.0096	
AB	1.31	1	1.31	0.0369	0.8531	
AC	157.21	1	157.21	4.42	0.051	
A²	1082.70	1	1082.70	30.43	0.0009	
B²	727.26	1	727.26	20.44	0.0027	
C²	529.96	1	529.96	14.90	0.0062	
AB²	864.40	1	864.40	24.30	0.0017	
Residual	249.04	7	35.58			
Lack of Fit	126.18	3	42.06	1.37	0.3725	Not significant

The Model F-value of 28.11 implies the model is significant. There is only a 0.01% chance that an F-value this large could occur due to noise. P-values less than 0.0500 indicate model terms are significant. In this case, A, B, C, AC, A², B², C², and AB² are significant model terms. Values greater than 0.1000 indicate the model terms are not significant.

The value of AB is 0.85; this means that this term can be neglected, and it has no effect on the conversion.

Conversion (Y) equation obtained from RSM in coded value.

5 $$\begin{array}{l}Y=10.88+14.66A+5.18B+7.44C+16.04{A}^{2}\\ \quad +13.14{B}^{2}-11.22{C}^{2}+20.79A{B}^{2}\end{array}$$

The conversion can be rewritten in the actual variables according to the following three equations.

6 $$\:A=\frac{T-90}{30}$$

7 $$B=\frac{c-8}{4}$$

8 $$\:\:\:C=\frac{t-3}{2}$$

conversion (Y) equation obtained from RSM in actual value (Eq. 9);

9 $$\begin{array}{l}Y = 92.48818 - 2.44575T - 11.84841C + 20.55092t + 0.017817{T^2}\\\quad + 0.729319{C^2} - 2.80475{t^2} + 0.001023T{C^2}\end{array}$$

**Fig. 4:**
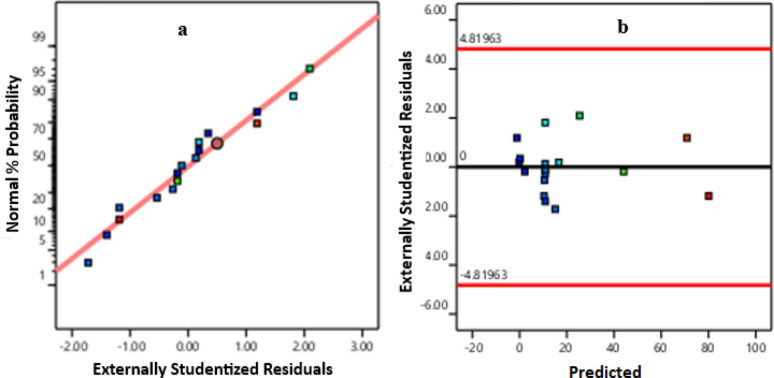
**a** Normal plot of residual, and** b** residuals versus predicted

Figure 4a, b displays the normal plot and the residuals versus predicted values, which validate the regression model and confirm the model’s robustness.

#### Effect of process parameters

**Fig. 5:**
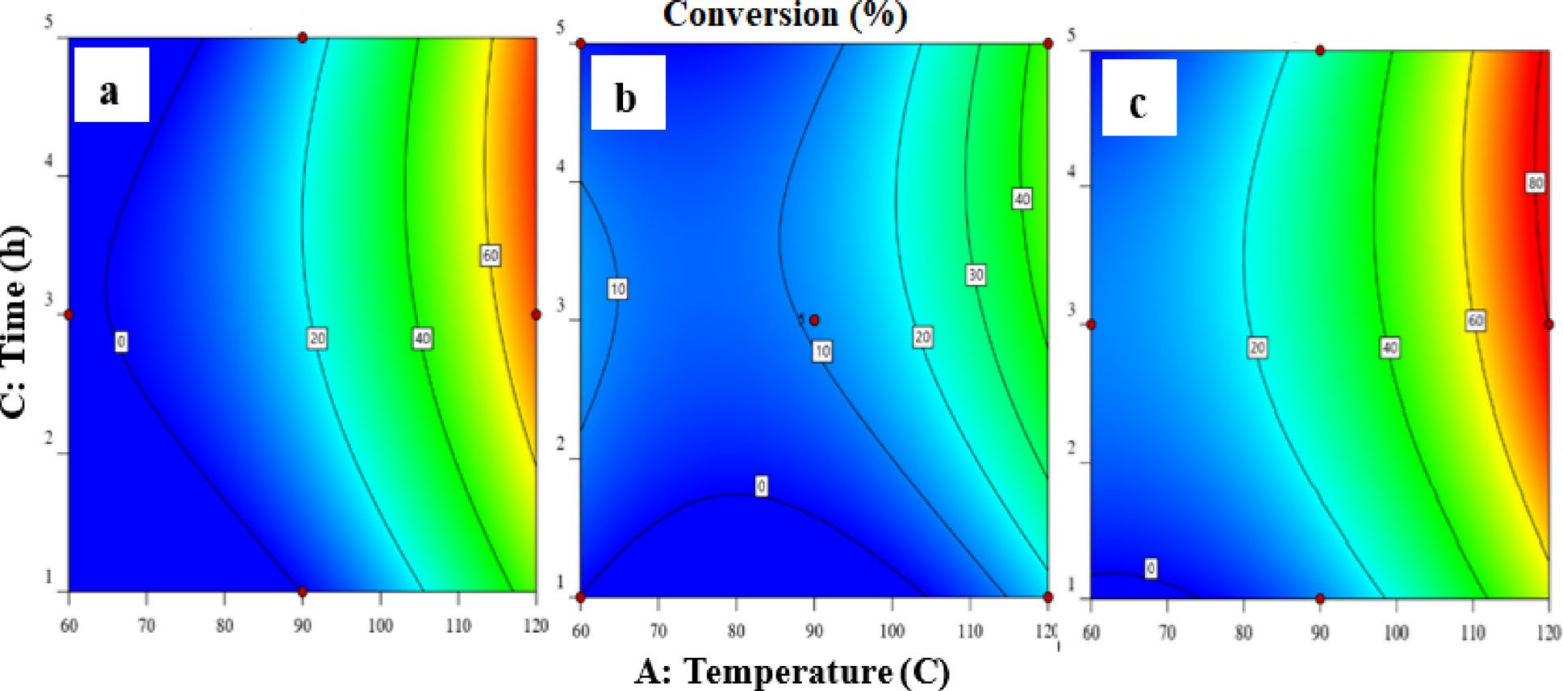
**a**,** b**,** c** The effect of temperature and time at minimum acid concentration (**a**, 4 M), medium (**b**, 8 M) and maximum (**c**, 12 M)

Figure 5a, b, and c indicates the effect of temperature and time on the conversion at minimum, medium, and maximum values of acid concentration. As shown in the figure, at minimum concentration, as temperature increases, the conversion increases, but the maximum value is lower than 70% as shown in Fig. 5a. At the maximum value of concentration, the conversion increases with increasing temperature and exceeds 80% as shown in Fig. 5c which refers to the interaction between the studied parameters. It can be noticed that increasing time is not effective at lower temperature and lower acid concentration but at maximum concentration Fig. 5c increasing time increases the conversion, especially at high temperature, which certifies the interaction between the studied parameters.

**Fig. 6:**
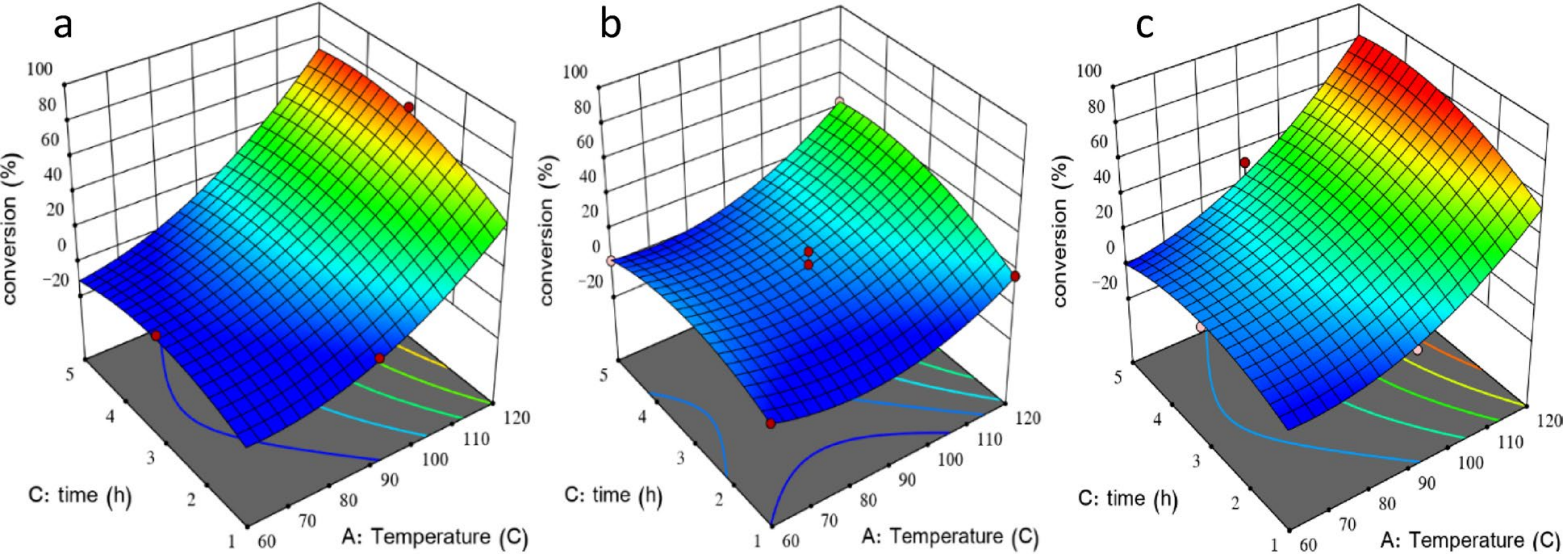
3D surfaces (**a**,** b**,** c**), the effect of temperature and time at minimum (**a**, 4 M), medium (**b**, 8 M) and maximum (**c**, 12 M) value of acid concentration

The 3D surfaces is displayed in Fig. 6.

#### Model optimization

**Fig. 7:**
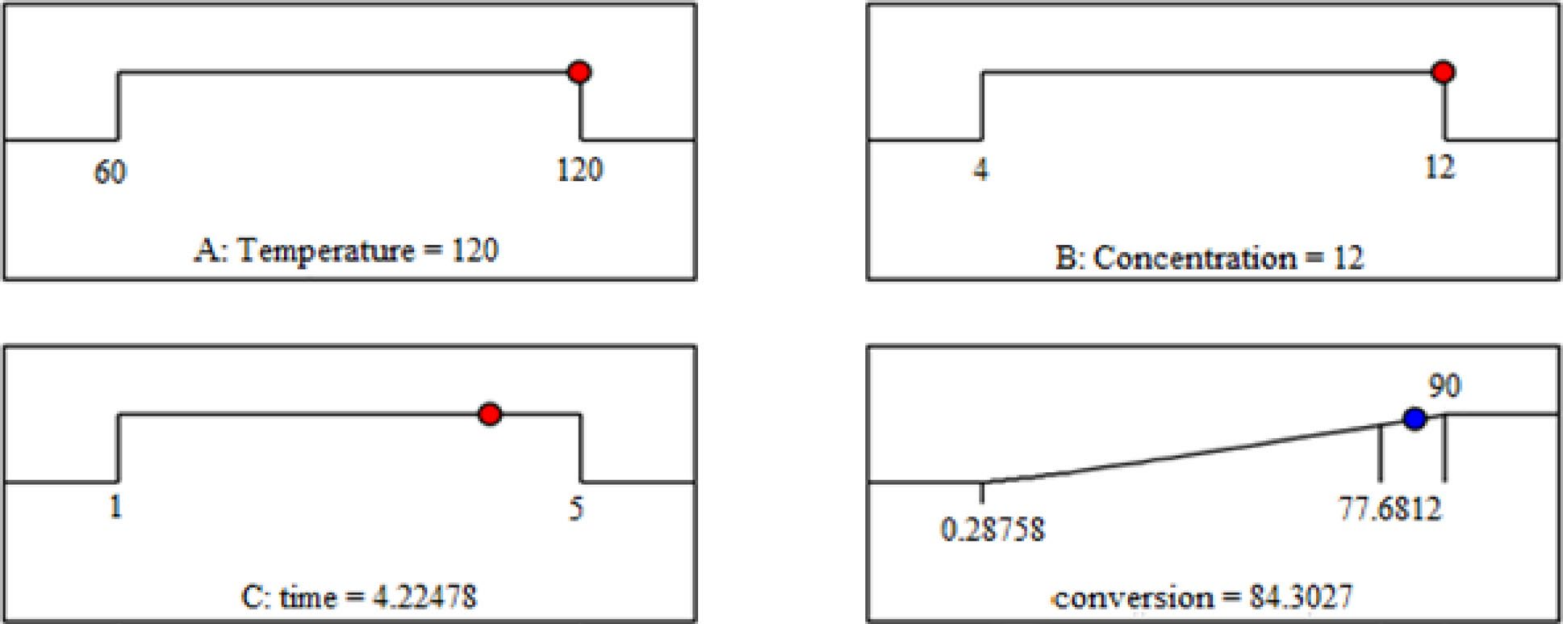
Optimization of studied parameters

RSM can be used to optimize the model and select the parameters needed to achieve the optimized Conversion of 84.3072% at a temperature 120 ℃, time 255 min, and concentration 12 M. The optimum condition was tested experimentally and the conversion of Titanium was 83.7% with an error of about 0.71% which means that the model is significant (Fig. 7).

San Yu, 2018 maintained a maximum extraction of titanium dioxide 78%, at an acid concentration (60%), temperature (150 °C), and leaching time (6)hrs [40].

### Characterization of prepared titanium dioxide

The prepared titanium dioxide after calcination undergoes thorough characterization. XRD, XRF, SEM, and EDX to paint a complete picture. XRD reveals the mineral phases present. XRF provides a precise chemical fingerprint, highlighting titanium content and any potential impurities. SEM provides information about the morphology (shape and size), surface features, and composition of the sample. EDX ensures that the sample is of the desired composition and identifies any potential impurities.

#### X-ray fluorescence

The XRF results of the product sample are shown in Table 6 : XRF analysis result states that the sample is mainly composed of titanium (96.4677% TiO_2_) and iron (2.9287% Fe_2_O_3_), which refers to the separation of most of the iron in hydrolysis step.

**Table 6:** XRF analysis

Compound formula	Percentage (wt%)	Compound formula	Percentage (wt%)
Na_2_O	0	Cr_2_O_3_	0
MgO	0.044818	MnO	0.057423
Al_2_O_3_	0.068627	Fe_2_O_3_	2.928571
SiO_2_	0.068627	Co_3_O_4_	0
P_2_O_5_	0.120448	ZnO	0
SO_3_	0.07563	ZrO_2_	0.022409
K_2_O	0	Nb_2_O_5_	0.112045
CaO	0.033613	Cl	0
TiO_2_	96.46779		

Kermania, et al. 2020 extracted titanium dioxide from illeminite with HCl (9 M), temperature 80 °C, and time (3 h). The purity of the produced synthesized-TiO_2_ was determined to be more than 90% with a mean particle size diameter of around 70 nm [39].

#### X-ray diffraction

**Fig. 8:**
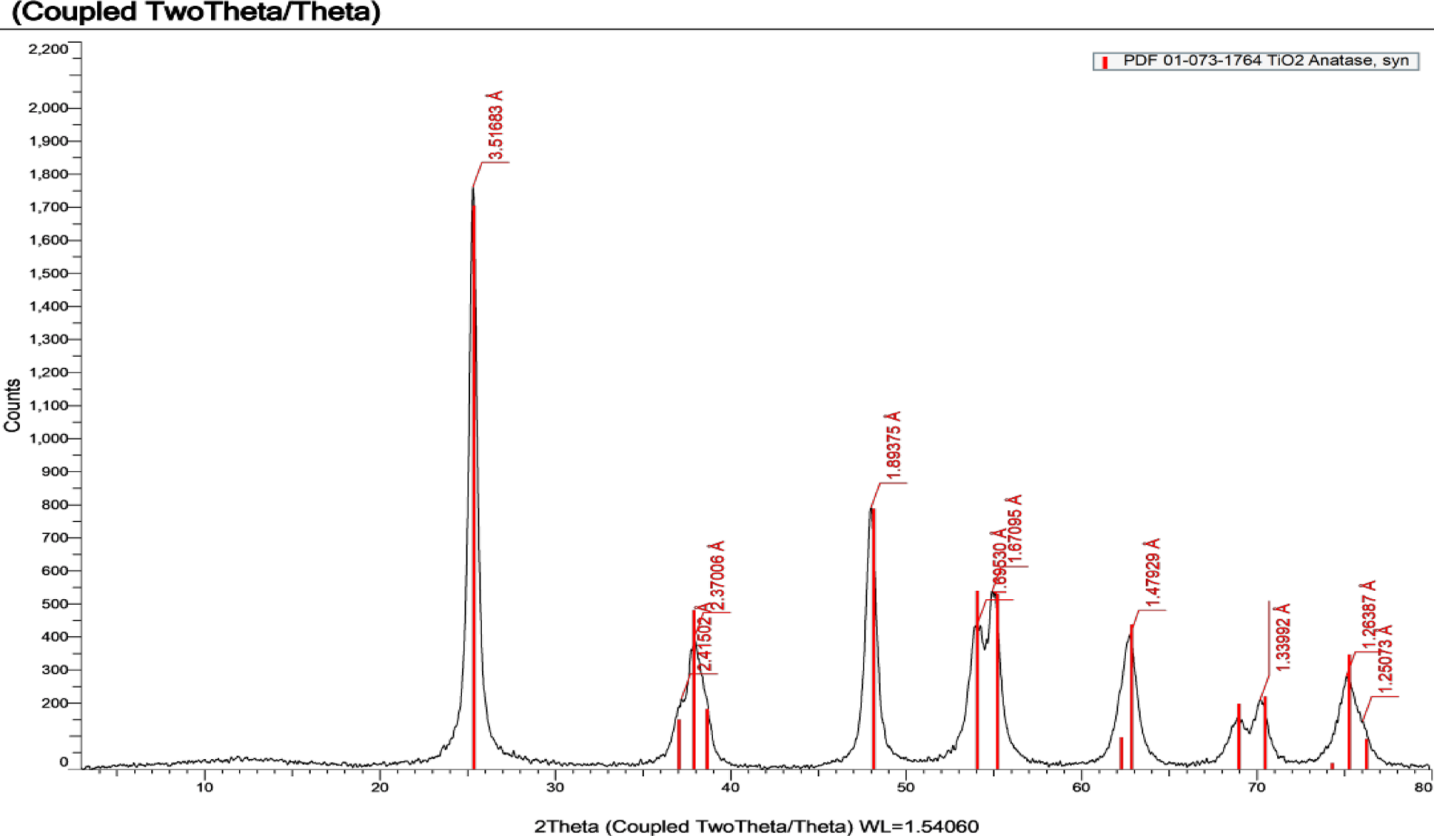
XRD of prepared titanium dioxide

The X-ray diffraction (XRD) results of the product titanium dioxide sample were used to indicate the crystal structure of the sample. The peaks in the grave indicate that the main phase of the sample is the anatase phase, as shown in Fig.8; Table 7 [37].

**Table 7:** XRD result colors

Color	Compound name	Formula	Percentage
	Anatase	Ti$\:{O}_{2}$	100%

There are no difficulties in discriminating between the anatase and the rutile phases since the first two reflection peaks are well separated (2θ = 25.28° for d101 of anatase vs. 2θ = 27.44° for d110 of rutile [41].

#### Scanning electron microscope

**Fig. 9:**
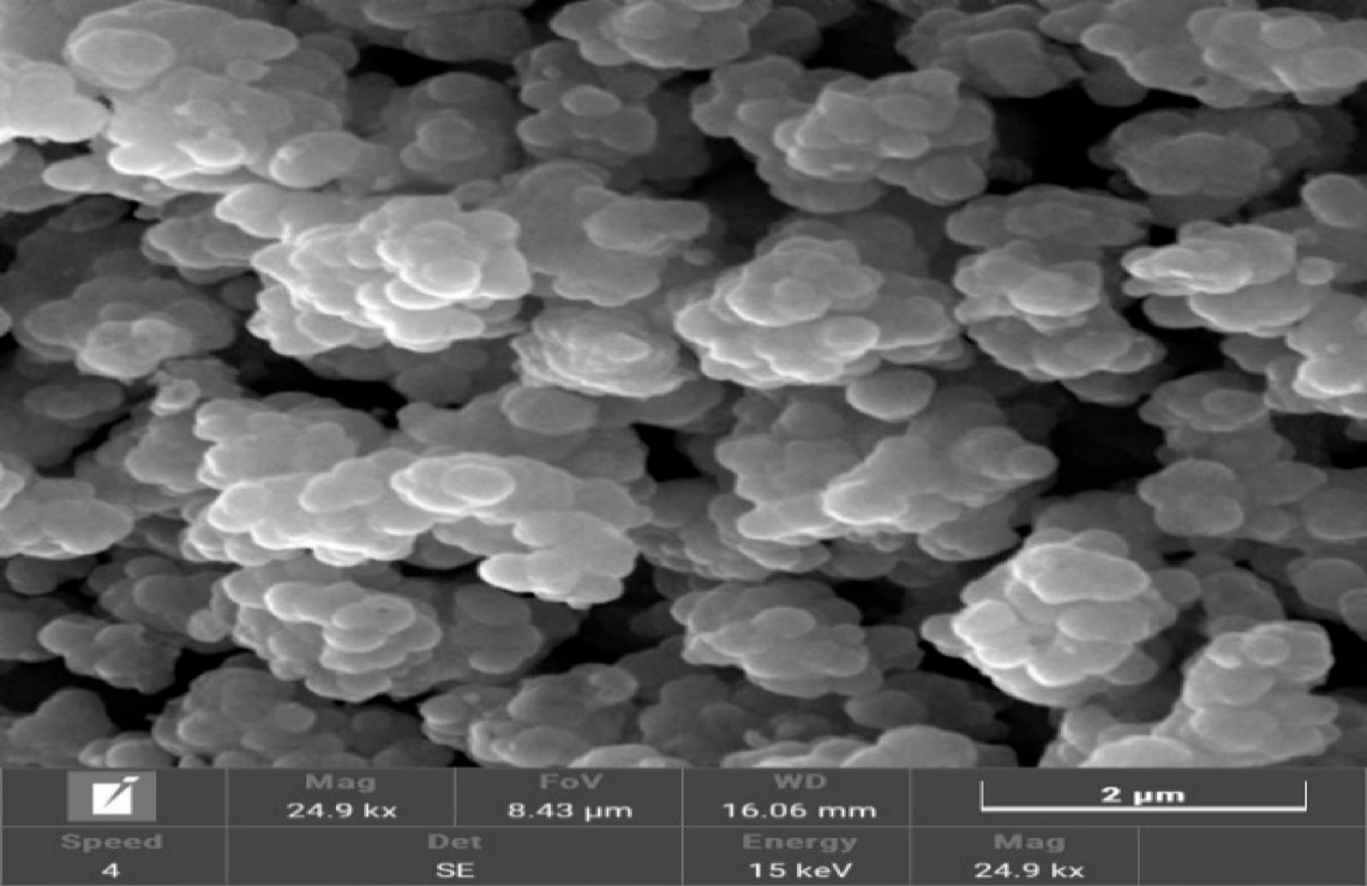
SEM of prepared titanium dioxide

The SEM image with 25kx magnification shows a high-magnification view of the titanium dioxide powder shape. As shown in Fig. 9 the images display that TiO_2_ has a uniform spherical morphology shape [42].

#### Energy dispersive X-ray analysis (EDX)

**Fig. 10:**
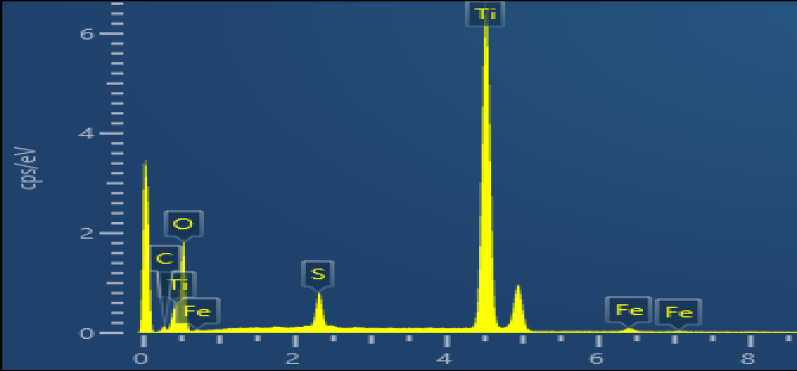
EDX results of prepared titanium dioxide

The EDX analysis confirms the presence of titanium dioxide (TiO_2_) in the sample. However, there is also evidence of some carbon, sulfur, and iron contamination. As it is shown in Fig. 10 and Table 8 Titanium (Ti) has the highest weight% at 61.31%, and Oxygen (O) is the second highest at 33.23%, which matches with the XRF results [42].

**Table 8:** EDX results of prepared titanium dioxide

Element	Weight%
C	1.89
O	33.23
S	2.25
Ti	61.31
Fe	1.33
Total	100.00

#### Size distribution

Particle size distribution was measured using Malvern MASTERSIZER 3000 (Laser Diffraction) to determine the average particle size of the prepared titanium dioxide sample. The calculated average particle size is about 78.82 nm. Because of its special photocatalytic and light-absorbing capabilities, titanium dioxide (TiO₂) nanoparticles, which have a size of 78.82 nanometers, find use in a variety of fields, such as solar energy, environmental cleanup, and medicine [43].

#### Fourier transform infrared spectroscopy

**Fig. 11:**
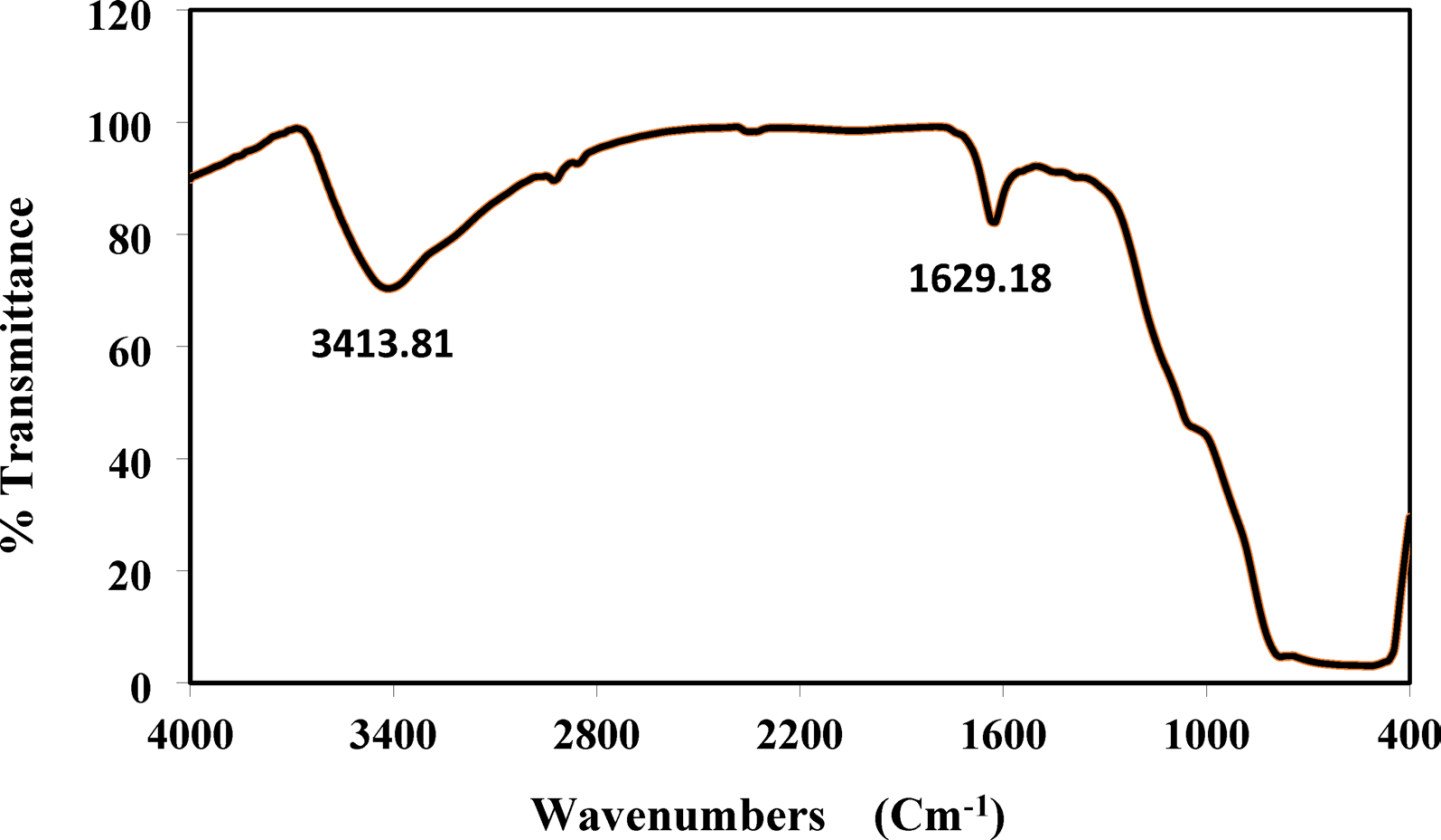
FTIR of prepared titanium dioxide

The FTIR spectrum of prepared TiO_2_ is shown in Fig. 11.

The FTIR study of TiO_2_ displays the characteristics of the formation of a high-purity product. From the FTIR spectrum, several peaks at 3413.81, 1628.18, 728.77, and 463.88 cm^− 1^ can be observed. Peaks at 3413.81, 1628.18 refer to the titanium dioxide sample has significant hydroxyl groups on the surface. The broad band from 1000 to 400 cm^− 1^ region is related to the Ti–O stretching and Ti–O–Ti bridging stretching modes. For the pure TiO_2_, the peaks appear at 463.88 and 728.77 cm^− 1^ are in the range of 400–800 cm^− 1^. The infrared absorption frequencies and the corresponding vibrational bonds are represented in Table 8 [44] (Table 9).

**Table 9:** FTIR absorption frequencies and corresponding vibrational bonds TiO_2_ [44]

Wavenumbers (cm^− 1^)	Type of bond
3391.72	O–H stretching
1628	O–H bending
728.77	Ti–O–Ti stretching
463.88	Anatase titania

## Conclusion

Ilmenite contains a high titanium dioxide concentration with a percentage reaching 43%. One of the common methods for titanium dioxide extraction from ilmenite is the sulfate process, which is recommended due to its extraction efficiencies, economics, and environmental impacts.

To study the raw material characteristics, XRF analysis shows ilmenite composition, which contains ~ 43% TiO_2_, XRD analysis confirms the presence of ilmenite with 89.7% in the sample, and screen analysis indicates the average particle size of ilmenite is 0.118 mm.

Factors such as temperature, time, and acid concentration affect the leaching conversion. The RSM approach was used to study the effect of these parameters on the reaction yield and determine the optimum conditions for maximum yield. The results were fitted to a reduced cubic model, with R^2^ of 0.973, adjusted R^2^ of 0.9385, and a P value is 0.0001, which indicates the model is significant.

Application of the RSM approach, for machine learning or AI-based predictive optimization process ML/AI, requires hundreds or thousands of data points to generalize well and avoid overfitting, which is not suitable for the present work, which aims to optimize the parameters through a definite selected range.

Conversion of ilmenite to TiO_2_ was optimized via RSM, achieving 84.3% at 120 °C, 255 min, and 12 M concentration. When the ideal conditions were examined experimentally, the model’s significance was demonstrated by the titanium conversion of 83.7% with an error of about 0.71%. Characterization of the prepared titanium dioxide sample was carried out using XRF, XRD, SEM, and EDX. XRF analysis confirmed a high concentration of titanium dioxide, with 96.4% by weight. XRD analysis identified the presence of the anatase phase, and SEM imaging revealed that TiO_2_ has a uniform spherical morphology shape. Finally, EDX analysis confirmed that the sample is mainly titanium dioxide. The average particle size of the prepared titanium dioxide using a particle size analyzer is about 78.82 nm. The suggested industrial applications of prepared titanium dioxide are: use in hydrogen production as a photocatalyst in water splitting devices, applications in high-performance coating, and in solar energy and photovoltaics.

## Data Availability

The datasets used and/or analysed during the current study are available from the corresponding author on reasonable request.
